# A Comparison of Intensity-Modulated Radiotherapy With Simultaneous Integrated Boost With Three-Dimensional Conformal Radiotherapy With Sequential Boost for Locally Advanced Cervical Cancer: A Dosimetric Study

**DOI:** 10.7759/cureus.32940

**Published:** 2022-12-25

**Authors:** Carlos Ivan Figueredo Negron, Oscar Gamboa Garay, Alexandra Pabón Girón, Jose Alejandro Esguerra Cantillo, Eduardo Guerrero Lizcano

**Affiliations:** 1 Radiation Oncology, Universidad Militar Nueva Granada, Instituto Nacional de Cancerología, Bogotá, D.C., COL; 2 Medical Physics, Instituto Nacional de Cancerología, Bogotá, D.C., COL; 3 Radiation Oncology, Instituto Nacional de Cancerología, Bogotá, D.C., COL

**Keywords:** intensity-modulated radiotherapy, sib, simultaneous integrated boost, imrt, cervical cancer

## Abstract

Objective

The aim of this study was to compare the dosimetric criteria between the intensity-modulated radiation therapy (IMRT) technique with a simultaneous integrated boost (SIB) and the three-dimensional conformal radiation therapy (3DCRT) technique with a sequential boost (SB) plans for patients with locally advanced cervical cancer (LACC).

Materials and methods

A retrospective dosimetric comparison was performed in 15 patients with locally advanced cervical cancer who had previously been treated with fractions of 1.8 Gy up to doses of 45, 54-55.8, and 59.4 Gy in 28-33 sessions using the three-dimensional conformal radiation therapy (3DCRT) technique with a sequential boost (SB) and who had a new planning that was made using the intensity-modulated radiation therapy (IMRT) technique with a simultaneous integrated boost (SIB) in 25 sessions. The conformity index, quality of coverage, homogeneity index, mean doses, maximum doses, and different organ at risk (OAR) dose constraints were calculated for the dosimetric comparison of treatment plans. Descriptive analysis was performed using measures of central tendency and dispersion for the quantitative variables and absolute and relative frequencies for the qualitative variables. The comparison was made using the Wilcoxon signed rank sum test for a type I error level of 0.05. The statistical software Stata 11 (StataCorp LLC, College Station, Texas, USA) was used in the analysis.

Results

The mean age of the patients was 52 years, 33% were stage IIIB, and 67% had squamous cell carcinomas. The conformity index was 0.74 and 0.46 (difference: 0.28; p<0.01), the quality of coverage was 0.84 and 0.94 (difference: -0.10; p<0.01), and the homogeneity index was 0.12 and 0.070 (difference: 0.052; p<0.01) for IMRT-SIB and 3DCRT-SB, respectively. When the mean doses of the OARs were compared, all were lower with the IMRT-SIB technique, with statistically significant differences in the rectum and bladder.

Conclusions

The IMRT-SIB technique achieves a greater conformation of the doses on the treatment volumes with a significant reduction of the doses on the bladder and rectum.

## Introduction

Cervical cancer is the fourth most frequent neoplasm in females worldwide. Approximately 85% of the global burden generated by this disease occurs in low- and middle-income countries [1]. Among the risk factors associated with cervical cancer are low socioeconomic status, early age at first sexual intercourse, multiple sexual partners, multiparity, the use of oral contraceptives, and infection by the human papillomavirus (HPV), where types 16 and 18 are present in 70% of cases of cervical cancer, this being the most common sexually transmitted infection among females, showing a higher prevalence in young females and those older than 65 years old [2].

The current standard of care for locally advanced stages is concurrent chemoradiotherapy followed by high-dose-rate (HDR) brachytherapy (BT) boost. It is considered the most effective treatment option for overall survival and progression-free survival [3-5]. This treatment scheme is recommended by different guidelines of international societies [6-8]. Treatment lasts approximately seven weeks and should not exceed eight weeks since prolonged treatment time decreases local control and overall survival by 1% for each additional day [9-12]. The technique of three-dimensional conformal radiation therapy (3DCRT) or intensity-modulated radiation therapy (IMRT) can be used to administer external beam radiotherapy [7]. Traditionally, to administer a boost to the primary tumor, parametrium [13], and lymph nodes, a sequential boost (SB) technique is used with the same dose per fraction, increasing the number of fractions, and larger total doses are achieved over the high-risk volumes mentioned above.

It is challenging to offer treatments with optimal duration and the least associated complications to achieve the best possible cancer outcome. Various options have been explored to improve the toxicity profile and reduce the duration of the treatment without affecting cancer outcomes. In this scenario, intensity-modulated radiotherapy has been shown to decrease toxicity at the genitourinary, gastrointestinal, and hematological levels, as well as improve the quality of life in phase II and III studies [14,15]. Additionally, simultaneous integrated boost (SIB) can be applied with this technique, which allows planning and irradiation of different target volumes at different dose levels in the same treatment session, making it possible to increase the dose to the boost volume while keeping the dose to the elective volume at a lower level. In the treatment of cervical cancer, IMRT-SIB is generally administered in 25 sessions, reducing the total treatment duration by 1-2 weeks, with dosimetric studies reporting results similar to those of sequential treatment, although with the heterogeneity of the dosimetric criteria evaluated [16-20].

The objective of this study is to carry out a retrospective dosimetric comparison in patients with locally advanced cervical cancer (LACC) previously treated with radiotherapy with sequential boosts in fractionation of 1.8 Gy up to doses of 45, 54-55.8, and 59.4 Gy, in 28-33 fractions, who underwent a new treatment plan using the IMRT-SIB technique in 25 fractions, with the same total doses. The conformity index, homogeneity index, and quality of coverage were evaluated for the planning target volumes (PTV). The mean dose, the maximum dose, and some volumetric criteria were evaluated in the organs at risk (OARs). These were also quantified in the sequential treatment plans previously administered.

## Materials and methods

The sample size for a paired mean difference was calculated using the information published by Sukhikh et al. [20] for bladder and rectum dose of the 2 cc of volume with the highest dose (D2cc), selecting the largest sample size that corresponds to the difference in bladder D2cc. For a difference of 4 Gy, a standard deviation (SD) of the difference of three, a power of 80%, and an alpha of 0.05, 13 patients are required. We included 15 patients with LACC previously treated with sequential boost, using the three-dimensional conformal radiation therapy (3DCRT) technique, who had undergone a planning CT scan with 3 mm thickness in our institution. For each patient, a new treatment planning was made using the IMRT-SIB technique. Planning target volumes were generated to receive 45 Gy (PTV_45: tumor, the cervix, the uterine body, the vagina, the parametrium, lymph nodes, and elective lymph node areas including the common, internal, and external iliac nodes, the obturator, and the presacral region), 54-55.8 Gy (PTV_54-55.8: tumor, the parametrium, and lymph nodes), and 59.4 Gy (PTV_59.4: lymph nodes). The bladder, rectum, sigmoid, bowel, kidneys, spinal cord, and femoral heads were defined as organs at risk (OARs). The IMRT-SIB plans were to be administered in 25 fractions, while the 3DCRT-SB plans were administered in 25, 30-31, and 33 fractions on PTV_45, PTV_54-55.8, and PTV_59.4 volumes, respectively.

For the PTVs, the conformity index was calculated according to the following formula [21]: $IC=\frac{(TV)^{2}_{PIV}}{TV\cdot PIV}$, where TV is target volume and PIV is prescription isodose volume. The ideal score is one; it means a perfectly conformal plan; therefore, the lower the score is, the less conformity the plan has.

In addition, the quality of coverage was calculated with the following formula [22]: $Q=\frac{I _{min}}{RI}$, where I_min_ is the minimum isodose at the PTV and RI is the prescription isodose.

The homogeneity index was also calculated for the PTVs, with the following formula, according to the International Commission on Radiation Units and Measurements (ICRU) Report 83 recommendation [23]: $HI=\frac{D _{2\%}-D _{98\%}}{D _{50\%}}$, where D_2%_ is the dose at 2% of the volume, D_98%_ is the dose at 98% of the volume, and D_50%_ is the dose at 50% of the volume. A homogeneity index closer to zero means a homogeneous treatment plan.

For the OAR, the mean and maximum doses and different constraints were measured, with which the dosimetric comparison of the treatment plans was elaborated.

Descriptive analysis was performed using measures of central tendency and dispersion for the quantitative variables and absolute and relative frequencies for the qualitative variables. The comparison was made using the Wilcoxon signed rank sum test for a type I error level of 0.05. We evaluated the normality of the distribution of the quantitative variables using the Shapiro-Wilk test. The statistical software Stata 11 (StataCorp LLC, College Station, Texas, USA) was used in the analysis.

## Results

The patients' characteristics are shown in Table 1. The mean age was 52 years. The most common International Federation of Gynecology and Obstetrics (FIGO) 2018 classification stage was stage IIIB, with five patients representing 33%, followed by stage IIB with four cases, stage IIIC1 with three cases, and stage IVA with three cases. The most frequent histological type was squamous cell carcinoma, which occurred in 10 patients, followed by adenocarcinoma and adenosquamous carcinoma, present in two patients each. In one patient, it was not possible to determine the histological subtype.

**Table 1:** Baseline characteristics of patients in the study SD: standard deviation; FIGO: International Federation of Gynecology and Obstetrics; EBRT: external beam radiation therapy; BT: brachytherapy; EQD2: equivalent dose in 2 Gy fractions

Variable	
Mean age in years (SD)	52 (12.2)
FIGO classification (number, %)	
IIB	4 (27)
IIIB	5 (33)
IIIC1	3 (20)
IVA	3 (20)
Histological subtype (number, %)	
Squamous cell carcinoma	10 (67)
Adenocarcinoma	2 (13)
Adenosquamous carcinoma	2 (13)
Without data	1 (7)
Mean EBRT dose in Gy (SD)	56.88 (2.53)
Mean BT dose in Gy (SD)	18.66 (13.7)
Mean EQD2 in Gy (SD)	82.37 (18.5)

The dosimetric parameters analyzed for the PTV are shown in Table 2. Statistically significant differences (p<0.01) were found between treatment plans with 3DCRT-SB compared to IMRT-SIB for PTV_54 in terms of quality of coverage (0.94 versus 0.84, respectively), conformity index (0.46 versus 0.74, respectively), and homogeneity index (0.07 versus 0.12, respectively).

**Table 2:** Dosimetric analysis by treatment technique 3DCRT-SB: three-dimensional conformal radiation therapy technique with a sequential boost; IMRT-SIB: intensity modulated radiation therapy technique with a simultaneous integrated boost; D0.03cc: dose of the 0.03 cc of volume with the highest dose; D50%: dose at 50% of the volume; D30%: dose at 30% of the volume; D15%: dose at 15% of the volume; V45Gy: cubic centimeters of the volume receiving 45 Gy; V20Gy: the percentage of the volume receiving 20 Gy; V10Gy: the percentage of the volume receiving 10 Gy

Variable	3DCRT-SB (95% CI)	IMRT-SIB (95% CI)	P-value
Mean quality of coverage	0.94 (0.92-0.97)	0.84 (0.77-0.91)	<0.01
Mean conformity index	0.46 (0.34-0.58)	0.74 (0.64-0.83)	<0.01
Mean homogeneity index	0.07 (0.05-0.09)	0.12 (0.09-0.15)	<0.01
Bladder			
Mean dose (Gy)	47.9 (43.2-52.5)	39.8 (36.2-43.3)	<0.01
Mean D50% (Gy)	48.7 (43.9-53.5)	40.1 (35.6-44.7)	<0.01
Mean D0.03cc (Gy)	60.7 (59.7-61.7)	61 (59.4-62.6)	0.7
Bowel			
Mean dose (Gy)	22.7 (21-24.3)	21 (19.2-22.5)	0.1
Mean D30% (Gy)	30.1 (26.8-33.4)	25.4 (22.5-28.4)	0.03
Mean D0.03cc (Gy)	56.9 (53.2-60.6)	57.7 (54-61.3)	0.75
Mean V45Gy (cc)	244.7 (106-383.3)	188.7 (90-287.4)	0.46
Left femoral head			
Mean dose (Gy)	23 (19.4-26.7)	19.5 (16.7-22.3)	0.11
Mean D15% (Gy)	34 (30-37.7)	30.2 (26-34.5)	0.15
Mean D0.03cc (Gy)	52.9 (48.8-57.1)	53 (50-56.2)	0.72
Mean V10Gy (%)	83.2 (70.5-95.9)	80 (65.8-94.2)	0.26
Mean V20Gy (%)	57.1 (38.9-75.4)	39 (28.4-49.8)	0.21
Right femoral head			
Mean dose (Gy)	23 (19.3-26.7)	19.2 (16.1-22.3)	0.1
Mean D15% (Gy)	33.5 (29.8-37.2)	27.9 (21.7-34.1)	0.1
Mean D0.03cc (Gy)	52.7 (49.3-56.1)	47.3 (39.3-55.4)	0.29
Mean V10Gy (%)	83.8 (71.7-95.8)	79.5 (66.8-92.3)	0.31
Mean V20Gy (%)	57.7 (39.7-75.8)	38.4 (26.3-50.6)	0.09
Kidneys			
Mean dose of the left kidney (Gy)	9.6 (6.9-12.3)	9.1 (5.9-12.3)	0.9
Mean dose of the right kidney (Gy)	13.9 (8-19.8)	10.7 (7.1-14.4)	0.27
Rectum			
Mean dose (Gy)	55.3 (48.9-55.6)	45.3 (41.4-49.1)	<0.01
Mean D50% (Gy)	53.5 (49.6-57.5)	46.9 (42-51.7)	0.03
Mean D0.03cc (Gy)	59.4 (58.1-60.7)	60 (58-61.9)	0.61
Spinal cord			
Mean maximum dose (Gy)	16.9 (9.5-24.3)	10.7 (4.6-16.8)	0.15

The 3DCRT-SB and the IMRT-SIB plans met all cases' prescription goals of V95%≥95%. However, the minimum dose in the PTV was found to be predominantly lower for IMRT-SIB plans than for 3DCRT-SB plans.

Regarding the dosimetric parameters evaluated in the organs at risk, which are shown in Table 2, a tendency to decrease the mean doses of the bladder, kidneys, bowel, rectum, and femoral heads was observed, achieving a statistically significant difference in the rectum and bladder (p<0.01). When the D50% was evaluated, a statistically significant (p<0.01) dose reduction was observed in the plans with IMRT-SIB, both in the rectum and in the bladder. The parameter D30% in the bowel was lower in the IMRT-SIB treatment plan, being statistically significant (p=0.03); the V45Gy showed a decrease without being statistically significant. A slight dose increase was observed when D0.03cc was evaluated in the bladder, rectum, bowel, and femoral heads. This increase was smaller in the left femoral head (0.1 Gy) and greater in the right femoral head (5.4 Gy), although it was not statistically significant in any case. The maximum dose to the spinal cord had an absolute reduction of 6.2 Gy; however, this was not significant (p=0.15). The femoral heads showed a decrease in D15%, V20Gy, and V10Gy with IMRT-SIB without achieving statistically significant differences.

**Figure 1:**
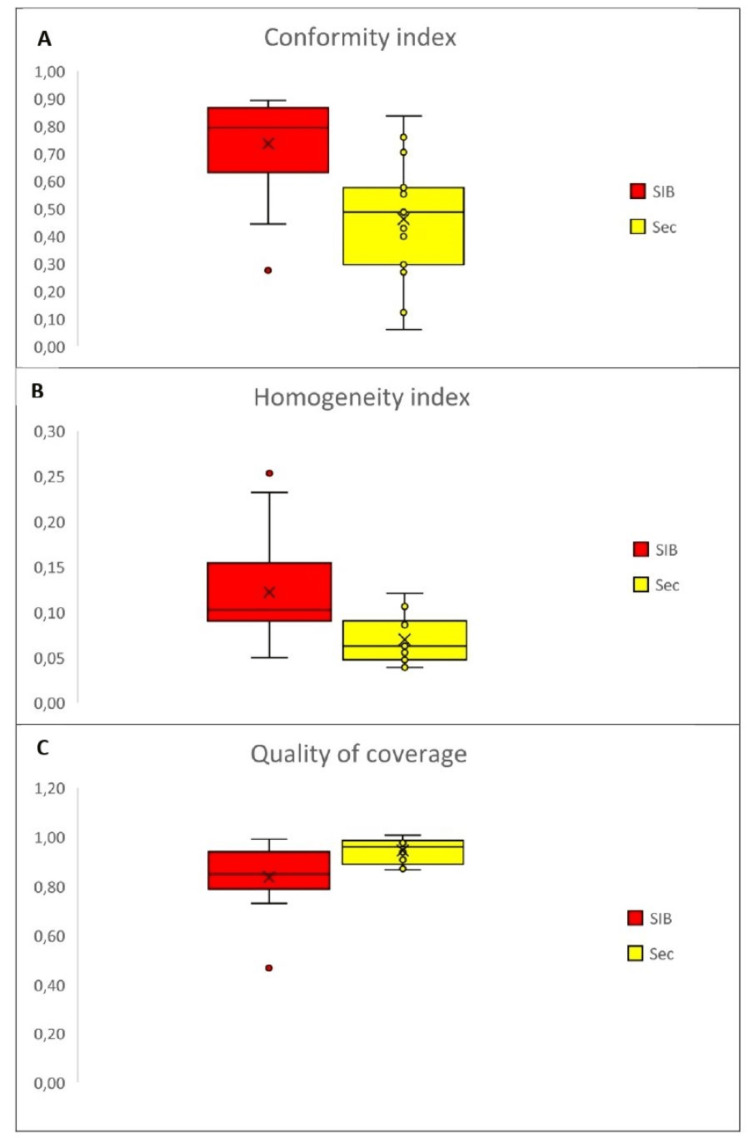
Box plot for conformity index (A), homogeneity index (B), and quality of coverage (C) by treatment technique SIB: simultaneous integrated boost; Sec: sequential boost

Figures 1A-1C show the box plot for the compared treatment techniques' conformity index, homogeneity index, and quality of coverage. It shows that the conformity index was higher for the technique with simultaneous integrated boost, the coverage index was higher, and the homogeneity index was lower for the sequential technique, with statistically significant differences.

## Discussion

The treatment of LACC represents a challenge for the radiation oncologist due to its necessary dose escalation to high-risk volume without an increase in dose to organs at risk, such as the rectum, sigmoid, bowel, and bladder. Fortunately, the advent of techniques such as IMRT has demonstrated their ability to optimize the dosimetric parameters of target volumes and decrease toxicity in OAR [14,15]. Additionally, IMRT allows the use of SIB, which makes it possible to decrease the total treatment time. Treatment time reduction is of great importance since it optimizes cancer center resources, which is especially relevant for low- and middle-income countries, where 85% of the cervical cancer burden worldwide is present and where, unfortunately, the infrastructure or equipment necessary for the treatment of these patients is not available.

The dosimetric comparison of our study found that the IMRT-SIB technique presents a better conformity index and a reduction in the mean doses to OAR, with statistically significant differences for the bladder and rectum compared to the sequential technique. The prescription goal of V95%≥95% (more than 95% of the prescribed dose delivered to more than 95% of the volume) was achieved in all plans. However, the limitations and potential difficulties inherent to IMRT in treating cervical cancer, such as organ movement, volume variability, dose inhomogeneity, and total dose, must be considered. This study's results align with those reported in the literature [18] and agree that the IMRT technique offers better conformity to target volume than 3DCRT. In addition, the dose distribution around the PTV was less homogeneous for the IMRT-SIB plans, as there was a lower dose in the PTV due to the intention of reducing high doses in areas where an intersection with the organs is to be protected.

In our study, all treatment plans were carried out using the IMRT-SIB technique, just as in the studies of Guerrero et al. [17] and Feng et al. [19]. However, other studies, such as those by Cheng et al. [18] and Vergalasova et al. [24], have used both IMRT and volumetric modulated arc therapy (VMAT) techniques, and Sukhikh et al. [20] even compared sequential to simultaneous integrated boost only with the VMAT technique. A comparison was made of 3DCRT-SB with the IMRT-SIB technique, using a 25-fraction scheme such as the studies of Guerrero et al. and Cheng et al. However, it differs in dose levels, these being 45, 54-55.8, and 59.4 Gy, which are higher than that used in other studies, similar to the dose levels assessed by Chino et al. [25], who evaluated doses of 2.4 Gy to 2.5 Gy in 25 sessions, up to a total of 60-70 Gy, demonstrating that dose escalation on pelvic and para-aortic lymph nodes is possible without an excessive increase in acute toxicity. In the present study, the doses applied to the parametrium and tumors were increased to 54-55.8 Gy, while the dose for lymph nodes was increased to 59.4 Gy. Despite these increases, a dose reduction in the rectum and bladder was achieved, as well as better conformity index.

Guerrero et al. [17] used radiobiological models to design new treatment regimens using IMRT-SIB in patients ineligible for intracavitary brachytherapy. They generated three IMRT-SIB plans with 25×1.8 Gy at the pelvic nodes and 25×2.4 Gy (60 Gy), 25×2.8 Gy (70 Gy), and 25×3.2 Gy (80 Gy) at the tumor site, with coverage that ranged from 94% to 95.5%, a result that is higher than that of our study that ranged from 77% to 91%. They also reported better preservation of the bladder and rectum with treatments of 60-70 Gy using SIB, compared to conventional treatments. This result is similar to that of the present study, where we observed a decrease in the dose of the OAR. It is important to clarify that the objective of this study is not to replace brachytherapy but to reduce the duration of treatment with external radiotherapy followed by a boost with HDR brachytherapy, with the aim of not exceeding the duration of the entire treatment by more than eight weeks.

Cheng et al. [18] compared SIB to parametrium using the VMAT or IMRT technique administered in 25 sessions with doses of 45 Gy (the pelvis) and 50 Gy (the parametrium) versus sequential boost with hemiblock of 5.4 Gy in three fractions. They reported a significant increase in the doses received by the OAR, which contrasts with our findings, where we observed a tendency to decrease the dose in the OAR that was significant in the bladder and rectum; however, we showed a tendency to increase D0.03cc that is not significant. They also found a better conformity index of the techniques with SIB of 0.680 and 0.687 compared to SB of 0.344, which was even better in this study at 0.74.

Feng et al. [19] carried out a dosimetric comparison of the sequential IMRT technique versus IMRT-SIB to evaluate dose escalation to avid lymph nodes in positron emission tomography (PET), which differs from our study given that the volumes in which the dose was escalated correspond to the tumor, parametrium, and lymph nodes. In that study, the authors found a reduction in the doses received by the organs at risk and hot spots in the PTV with similar coverage, results that resemble the ones found in our study.

Arnesen et al. [26] evaluated in patients with LACC with large and asymmetric tumors, who were previously treated and who had 18F-fluorodeoxyglucose positron emission tomography (18F-FDG PET) available before treatment, the use of short-course SIB with 2.8 Gy per fraction for the 10 first fractions at MTV50 (tumor volume defined by auto-segmentation using 50% SUVmax as threshold). Then, they continued with 1.8 Gy per fraction in the remaining 18 fractions with intensity-modulated proton therapy (IMPT) or VMAT technique, and it was compared with a scheme of 28 fractions up to a total dose of 50.4 Gy at the pelvic level followed by brachytherapy concomitantly with cisplatin, finding a marginal increase in the dose to the OAR for both modalities with SIB, where the majority of patients' dose volume histogram (DVH) parameters showed a mean difference below 2%. It is important to keep in mind that this study also included plans using both photons and protons, despite which a dose increase in OAR was observed, contrasting with the findings of our study where a tendency toward dose decrease in the OAR was evidenced, particularly in the rectum and bladder. The number of planned sessions was also different, as was the time when SIB was used; in our study, it was administered in all the sessions. Although the study conducted by Arnesen et al. differs from ours in several ways, it suggested a possible benefit of using SIB to achieve more significant tumor volume reduction in large and irregular tumors before receiving brachytherapy.

The study has limitations as it is a retrospective study, in which the planning of the IMRT-SIB treatment was carried out after having administered the radiotherapy treatment sequentially, which was the standard of care in our institution at the time that the patients were treated. Therefore, prospective studies are required to compare sequential planning techniques with SIB to determine if reducing the dose in the rectum and bladder with the IMRT-SIB technique decreases the incidence and severity of toxicity associated with radiotherapy, maintaining the effectiveness of treatment for local control of the disease.

This study provides dosimetric evidence for the IMRT-SIB technique, which may be a valid alternative to the 3DCRT-SB technique in treating females with LACC, achieving greater conformity, and reducing doses to OAR, which may translate into a decrease in toxicity and better local control in addition to the advantage of reducing the treatment application time in a pathology where it must be administered in seven weeks.

## Conclusions

Comparing the radiotherapy 3DCRT-SB technique with IMRT-SIB technique in the treatment of locally advanced cervical cancer, after analyzing the 15 plans made, we can conclude that this technique offers adequate coverage and homogeneity and allows an improvement in conformity for treatment volumes. In addition, it allows a general reduction in the dose received by the pelvic organs at risk while reducing the total treatment time, offering advantages to patients who can complete their treatment in less time and to centers of treatment, allowing optimization of resources. This is a promising technique, even though, to date, several retrospective and prospective observational studies show a similar toxicity profile; therefore, it is necessary to carry out clinical trials that evaluate clinical outcomes and the quality of life in this group of patients.
